# Indirect effect of oral azithromycin on the gut resistome of untreated children: a randomized controlled trial

**DOI:** 10.1093/inthealth/ihaa029

**Published:** 2020-06-17

**Authors:** Catherine E Oldenburg, Armin Hinterwirth, Lee Worden, Ali Sié, Clarisse Dah, Lucienne Ouermi, Boubacar Coulibaly, Lina Zhong, Cindi Chen, Kevin Ruder, Thomas M Lietman, Jeremy D Keenan, Thuy Doan

**Affiliations:** Francis I Proctor Foundation, 513 Parnassus Avenue, University of California, San Francisco, San Francisco, CA 94143, USA; Department of Ophthalmology, University of California, San Francisco, San Francisco, CA, USA; Department of Epidemiology & Biostatistics, University of California, San Francisco, San Francisco, CA, USA; Francis I Proctor Foundation, 513 Parnassus Avenue, University of California, San Francisco, San Francisco, CA 94143, USA; Francis I Proctor Foundation, 513 Parnassus Avenue, University of California, San Francisco, San Francisco, CA 94143, USA; Centre de Recherche en Santé de Nouna, Rue Namory Keita, Nouna, Burkina Faso; Centre de Recherche en Santé de Nouna, Rue Namory Keita, Nouna, Burkina Faso; Centre de Recherche en Santé de Nouna, Rue Namory Keita, Nouna, Burkina Faso; Centre de Recherche en Santé de Nouna, Rue Namory Keita, Nouna, Burkina Faso; Francis I Proctor Foundation, 513 Parnassus Avenue, University of California, San Francisco, San Francisco, CA 94143, USA; Francis I Proctor Foundation, 513 Parnassus Avenue, University of California, San Francisco, San Francisco, CA 94143, USA; Francis I Proctor Foundation, 513 Parnassus Avenue, University of California, San Francisco, San Francisco, CA 94143, USA; Francis I Proctor Foundation, 513 Parnassus Avenue, University of California, San Francisco, San Francisco, CA 94143, USA; Department of Ophthalmology, University of California, San Francisco, San Francisco, CA, USA; Department of Epidemiology & Biostatistics, University of California, San Francisco, San Francisco, CA, USA; Francis I Proctor Foundation, 513 Parnassus Avenue, University of California, San Francisco, San Francisco, CA 94143, USA; Department of Ophthalmology, University of California, San Francisco, San Francisco, CA, USA; Francis I Proctor Foundation, 513 Parnassus Avenue, University of California, San Francisco, San Francisco, CA 94143, USA; Department of Ophthalmology, University of California, San Francisco, San Francisco, CA, USA

**Keywords:** antimicrobial resistance, azithromycin, randomized controlled trial

## Abstract

**Background:**

Antibiotic use by one individual may affect selection for antimicrobial resistance in close contacts. Here we evaluated whether oral antibiotic treatment of one child within a household affected the gut resistome of an untreated cohabiting child.

**Methods:**

Households with at least two children <5 y of age were randomized in a 1:1 fashion to a 5d course of azithromycin or placebo. To evaluate indirect effects of azithromycin treatment on the gut resistome, we randomly assigned one child in the house to azithromycin and one to placebo. In placebo households, each child received placebo. We performed DNA sequencing of rectal swabs collected 5 d after the last antibiotic dose. We estimated risk ratios for the presence of genetic resistance determinants at the class level using modified Poisson models for children in azithromycin households compared with placebo households and assessed the composition of the resistome using permutational analysis of variance (PERMANOVA).

**Results:**

Of 58 children (n = 30 azithromycin households, n = 28 placebo households) with post-treatment rectal swabs, genetic resistance determinants were common but there was no significant difference at the class (p = 0.54 for macrolides) or gene (p = 0.94 for structure by PERMANOVA, p = 0.94 for diversity) level between untreated children in azithromycin households compared with placebo households.

**Conclusions:**

The results are encouraging that one child's antibiotic use may not influence the resistome of another child.

**Trial registration:** ClinicalTrials.gov NCT03187834.

## Introduction

Antibiotic consumption has been shown to select for antibiotic resistance and lead to expansion of the gut resistome at the individual and community levels.^1,2^ The resistome is defined as the collection of resistance gene determinants in a given environment. As the gut is the largest reservoir of pathogens in humans, understanding how antibiotic administration affects the resistome in populations is important to predict potential unintended consequences of increasing antibiotic use. Paediatric antibiotic use is increasing in many regions globally.^3^ Prophylactic mass drug administrations with azithromycin in childhood are being considered in some regions with exceptionally high mortality rates.^4^ Such programs would almost certainly be targeted to a subset of the population (e.g. preschool children). Analysis of both phenotypic and genotypic resistance following mass drug administration with azithromycin in treated individuals has generally shown increases in macrolide resistance.^5,6^ However, whether antibiotic treatment of an individual results in selection for genetic resistance determinants in untreated close contacts is unclear.

Previous work has shown no evidence of an indirect effect of sibling antibiotic use on the composition of the gut microbiome.^7^ However, a study of hospitalized patients showed that antibiotic use by hospitalized patients led to increased risk of *Clostridium difficile* infection in subsequent occupants of the same bed.^8^ Transfer of antibiotic resistance genes between cohabiting children could occur via faecal–oral transmission via direct physical contact or shared vectors, such as eating utensils or latrines. The objective of this study was to determine whether antibiotic treatment of one child in a household led to a change in the gut resistome in an untreated cohabiting child compared with the effect of placebo on an untreated child's resistome in a randomized controlled trial of the effects of antibiotic treatment on the gut microbiome.^9^ We evaluated differences in genetic resistance determinants to macrolides in placebo-treated children in azithromycin- compared with placebo-treated households as well as the resistome in its totality, including specific antibiotic classes and the overall composition of the resistome.

## Methods

### Study setting

This study took place in two communities in the Nouna Health and Demographic Surveillance Site (HDSS) in northwestern Burkina Faso in July 2017.^10^ The area is rural and agrarian. Healthcare for children <5 y of age is free in Burkina Faso and delivered via nurse-led primary care clinics. Antibiotic consumption is not uncommon among preschool children and the most commonly prescribed antibiotics are amoxicillin, co-trimoxazole and erythromycin.^11^

### Trial methods

**Figure 1.**
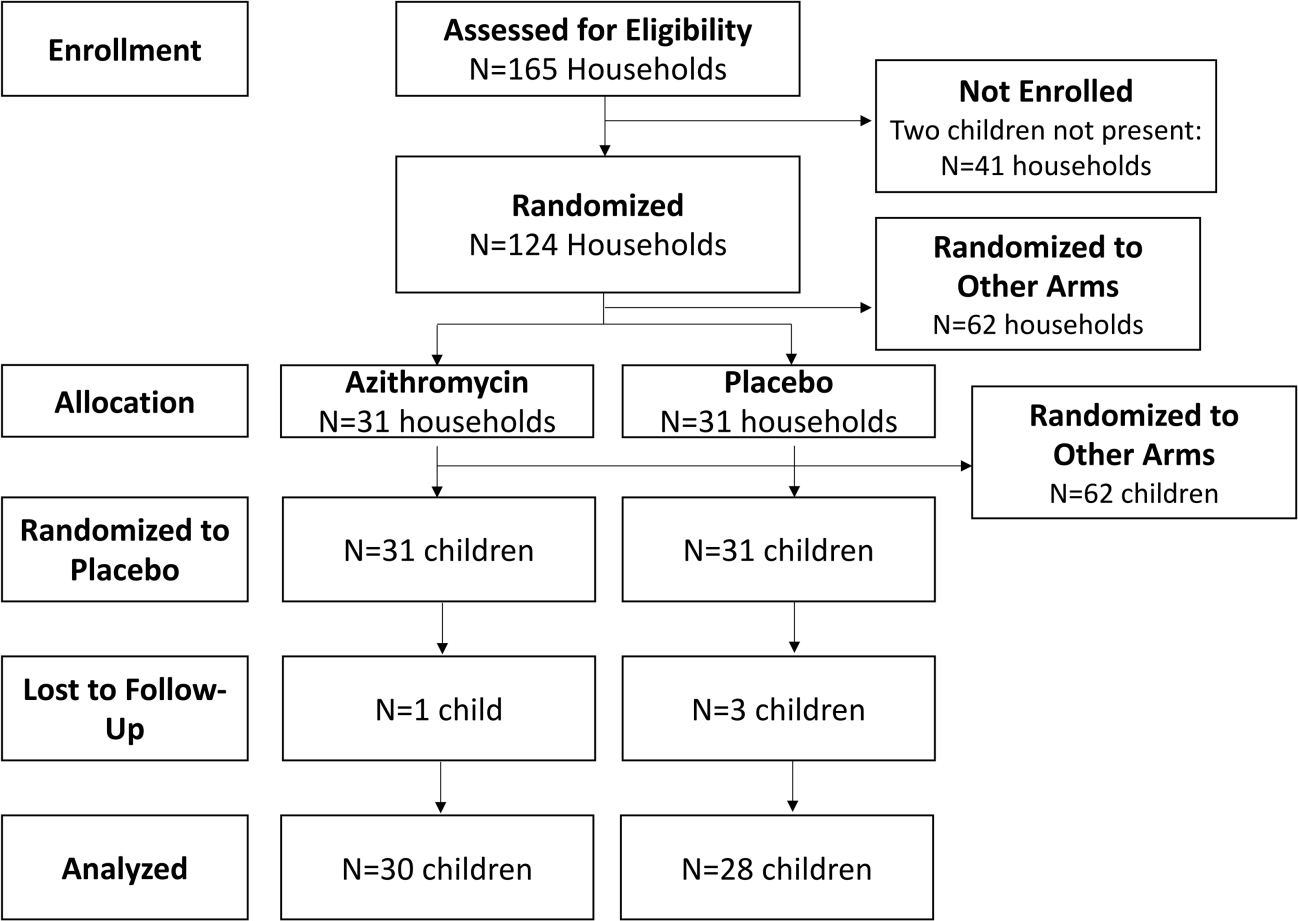
Consolidated Standards of Reporting Trials diagram for participants included in the study and analysis.

Complete methods for the trial have been previously published.^2,7,9^ Households with at least two children <5 y of age in residence were eligible for inclusion in the study and two children 6–59 months of age were included from each participating household. Eligible households that agreed to participate were randomized in a 1:1:1:1 fashion to a 5d course of amoxicillin, azithromycin, co-trimoxazole or placebo. Within each household, one child was randomized to the household's antibiotic assignment and one child to placebo. In placebo households, both children received placebo, and one child was randomly assigned to serve as the control for the antibiotic-treated child in the antibiotic households and one as the control for the placebo-treated child in the antibiotic households. The present report is limited to children randomized to placebo in azithromycin households and to the placebo-treated child control in placebo households (Figure 1). A direct effect of antibiotics on the resistome was only observed among children receiving azithromycin compared with placebo; we were unable to process samples from the other study arms due to cost constraints. The placebo consisted of a powdered milk and sugar solution in bottled water, made fresh each day. All treatments were administered in opaque syringes and administered via a central distribution point in each study community. All treatments were directly observed by study staff.

Rectal samples were obtained 5 d after the last treatment dose was administered. Swabs were placed immediately in a Stool Nucleic Acid Collection and Transport Tube with Norgen Stool Preservative (Norgen Biotek, Thorold, ON, Canada). Samples were collected at the ambient temperature in the field and stored at −80°C in Nouna until they were shipped to San Francisco (CA, USA). Samples were de-identified and processed in a random order. All laboratory personnel were masked to the child and household treatment assignment.

### Laboratory methods

DNA was extracted using the Norgen stool DNA isolation kit per the manufacturer's instructions. DNA sequencing was performed as previously described.^1,2^ Briefly, sequencing libraries were prepared using the NEBNext Ultra II DNA Library Prep Kit (New England Biolabs, Ipswich, MA, USA) and then amplified with 10 polymerase chain reaction cycles. Samples were sequenced on the NovaSeq system (Illumina, San Diego, CA, USA) using 150-nucleotide paired-end sequencing. Human sequencing reads were removed and the remaining non-host read pairs were then passed onto Centrifuge (version 1.0.3) to align to the entire National Center for Biotechnology Information non-redundant collection.^1^ Non-host reads were aligned to the MEGARes reference antimicrobial database using the Burrows–Wheeler aligner with default settings. Only antimicrobial resistance determinants (ARDs) with a gene fraction >80% were identified as present in the sample and included for further analyses.^2^ Each identified ARD was classified at the class and gene level using the Resistome Analyzer (https://github.com/cdeanj/resistomeanalyzer).

### Statistical methods

The sample size was determined based on the primary outcome, which was gut microbial diversity.^9^ A sample size of 30 children per arm was estimated to provide at least 80% power to detect a 1.5-unit difference in Simpson's α diversity. The indirect effect of a cohabiting child's antibiotic treatment on an untreated child was assessed by comparing selection for genetic resistance determinants at both the class and gene levels between placebo-treated children in azithromycin households and placebo-treated children in placebo households. We estimated risk ratios (RRs) for the presence of genetic resistance determinants at the class level using modified Poisson models for children in azithromycin compared with placebo households. As a sensitivity analysis, we included the child's age and recent antibiotic use as covariates in the model. At the class level, we compared post-treatment normalized reads for abundance in children in azithromycin compared with placebo households. To determine the community structure differences, we performed permutational analysis of variance (PERMANOVA) on the L^2^-norm (Euclidean) distance between treatment groups. Inverse Simpson's diversity was used to evaluate the resistome at the class and gene levels and converted to the effective number per sample. These diversity values can be considered as a measure of the richness of the resistome. A two-sided permutation test on differences in α diversity of the two treatment groups were used for the diversity measures. The number of permutations was 10 000 in all cases. All analyses were conducted in R version 3.4.3 (R Foundation, Vienna, Austria).

## Results

**Figure 2.**
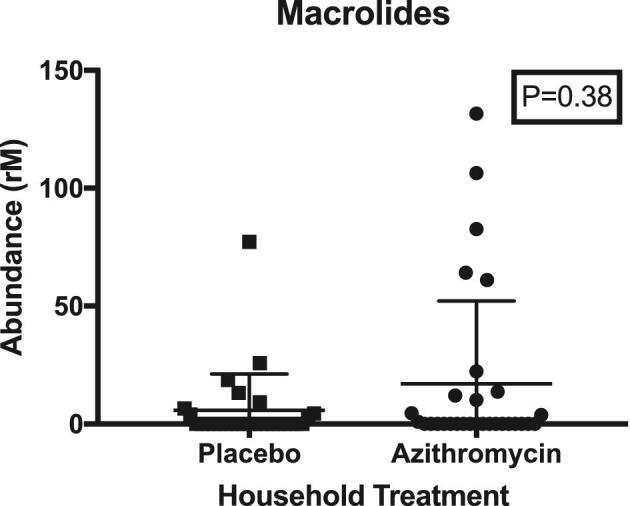
Abundance (normalized reads) of macrolide resistance genes in children treated with placebo living in households with another child treated with placebo or azithromycin.

Of 61 children randomized to placebo in households randomized to azithromycin or placebo, rectal swabs at 5d post-treatment were collected from 58 children (95%; n = 30 from azithromycin households and n = 28 from placebo households; Figure 1). Of these 58 children, >95% of their cohabiting siblings finished their respective treatment course.^12^ Approximately two-thirds of enrolled children were female and the median age was 20 months (Table 1). Resistance genetic determinants to multiple classes of antibiotics were common in the gut, particularly tetracyclines, trimethoprim and beta-lactams (Table 2). Treatment with azithromycin in one child did not result in an acute increase in macrolide resistance determinants in another child who is cohabiting in the same household (RR 1.24 [95% CI 0.62 to 2.49], p = 0.54; Table 2, Figure 2). Similarly, no differences were seen between treatment arms for other antibiotic classes (Table 2). Results were robust to inclusion of age and recent antibiotic use as covariates. There was no statistically significant difference in the structure of the gut resistome in cohabiting children in households treated with azithromycin compared with placebo (Euclidean PERMANOVA, p = 0.94). In addition, the diversity of the resistome is similar between treatment groups at the antibiotic class level (inverse Simpson's index 1.7 [95% CI 1.4 to 2.0] vs 2.0 [95% CI 1.6 to 2.7], p = 0.94) or at higher resolution at the gene level (inverse Simpson's index 9.5 [95% CI 7.6 to 12.3 vs 12.5 [95% CI 12.7 to 17.3], p = 0.94).

**Table 1.** Baseline characteristics of untreated children in azithromycin and placebo households

		
	Azithromycin (n = 31)	Placebo (n = 30)
Age (months), median (IQR)	42 (33–46)	39 (35–46)
Female, n (%)	19 (61.3)	20 (66.7)
Antibiotic use in the past 30 days, n (%)	1 (3.2)	5 (16.7)

**Table 2.** Prevalence of and RRs for the presence of class-level genetic resistance determinants in children in households randomized to azithromycin compared with placebo

Antibiotic class of genetic resistance determinants	Azithromycin household (n = 30), n (%)	Placebo household (n = 28), n (%)	RR (95% CI)	p-Value
Macrolide	12 (40)	9 (32)	1.24 (0.62 to 2.49)	0.54
Beta-lactams	26 (87)	21 (75)	1.55 (0.89 to 1.49)	0.27
Sulphonamides	6 (20)	6 (21)	0.93 (0.34 to 2.56)	0.93
Trimethoprim	19 (63)	17 (61)	1.04 (0.70 to 1.56)	0.84
Aminoglycosides	8 (27)	10 (36)	0.75 (0.34 to 1.62)	0.46
Fluoroquinolones	10 (33)	11 (39)	0.85 (0.43 to 1.68)	0.64
Tetracyclines	28 (93)	23 (82)	1.14 (0.93 to 1.38)	0.20
Multidrug resistance	12 (40)	18 (64)	0.62 (0.37 to 1.04)	0.07

## Discussion

In this randomized controlled trial, we found no statistically significant evidence that antibiotic treatment of one child leads to an increase in selection for genetic resistance determinants or expansion of the resistome in an untreated cohabiting child. Previous studies of antibiotic use have found evidence of rapid and dramatic expansion of the resistome in treated children.^2^ As mass azithromycin distribution programs for prevention of child mortality are being considered, a major concern is selection for macrolide and other antibiotic class resistance, including transfer of resistance to untreated individuals.^13,14^ A spillover effect of selection for resistance to untreated individuals would be concerning, because it may indicate the potential for wider spread of resistance even in populations where only a subset are treated (e.g. younger children). In this study there was a small increase in macrolide resistance in children in households where a cohabiting child had received azithromycin vs placebo, however, this difference was not statistically significant. Therefore this study did not provide evidence that treatment of one child in a household affects the resistome of an untreated child within that household.

In addition to no significant evidence of selection for genetic resistance determinants at the class level, there was no significant evidence of expansion of the resistome at the gene level. Although azithromycin treatment was previously shown to lead to selection for macrolide resistance determinants compared with placebo, there was no difference in richness in azithromycin-treated children compared with placebo-treated children nor was there a difference in the composition of the genetic resistance determinant.^2^ A previous analysis found no difference in diversity or composition of the gut microbiome in cohabiting children living with an azithromycin-treated child compared with placebo.^7^ Taken together, these results do not provide evidence of a spillover effect of azithromycin use on the gut microbiome and resistome.

This analysis should be considered in the context of several limitations. This study was powered for detection of a direct effect of antibiotic use on the gut microbiome. Any indirect effect of antibiotic use would likely be smaller than a direct effect and thus this study was likely underpowered to detect small differences in the resistome. CIs were fairly wide for most outcomes, and further studies with larger sample sizes are required to evaluate whether there is a small effect of household antibiotic use on the resistome of untreated children. Samples were collected 5 days after the last antibiotic dose. Although treatment itself may rapidly lead to changes in the resistome, indirect effects may take longer to manifest. Macrolide resistance carriage likely persists for >5 d in the gut,^15^ and this analysis likely underestimates total transmission of resistance genes between children. This study does not provide data on longer-term spillover effects. Finally, the results of this study are likely not generalizable outside of the study's setting. Results may be affected by background antibiotic use, local pathogenic and commensal bacteria and patterns of interaction between cohabiting children and their environment and therefore may not translate to other settings.

In summary, we were unable to detect an indirect effect of paediatric antibiotic use on the resistome of an untreated cohabiting child. These results are encouraging that one child's antibiotic use may not influence the resistome of another child, but the CIs were fairly wide and indicate a lack of precision in the estimates. However, any indirect effect of systemic antibiotic use between children on the paediatric resistome is likely small.
